# Remote imaging of single cell 3D morphology with ultrafast coherent phonons and their resonance harmonics

**DOI:** 10.1038/s41598-019-42718-5

**Published:** 2019-04-23

**Authors:** Liwang Liu, Alexis Viel, Guillaume Le Saux, Laurent Plawinski, Giovanna Muggiolu, Philippe Barberet, Marco Pereira, Cédric Ayela, Hervé Seznec, Marie-Christine Durrieu, Jean-Marc Olive, Bertrand Audoin

**Affiliations:** 1 University of Bordeaux, CNRS UMR 5295, I2M, F-33400 Talence, France; 2 University of Bordeaux, CNRS UMR 5248, Bordeaux INP, CBMN, F-33600 Pessac, France; 3 University of Bordeaux, CNRS UMR 5797, CENBG, F-33170 Gradignan, France; 4 University of Bordeaux, CNRS UMR 5218, IMS, F-33400 Talence, France

**Keywords:** Acoustics, Photoacoustics, Imaging techniques, Imaging

## Abstract

Cell morphological analysis has long been used in cell biology and physiology for abnormality identification, early cancer detection, and dynamic change analysis under specific environmental stresses. This work reports on the remote mapping of cell 3D morphology with an in-plane resolution limited by optics and an out-of-plane accuracy down to a tenth of the optical wavelength. For this, GHz coherent acoustic phonons and their resonance harmonics were tracked by means of an ultrafast opto-acoustic technique. After illustrating the measurement accuracy with cell-mimetic polymer films we map the 3D morphology of an entire osteosarcoma cell. The resulting image complies with the image obtained by standard atomic force microscopy, and both reveal very close roughness mean values. In addition, while scanning macrophages and monocytes, we demonstrate an enhanced contrast of thickness mapping by taking advantage of the detection of high-frequency resonance harmonics. Illustrations are given with the remote quantitative imaging of the nucleus thickness gradient of migrating monocyte cells.

## Introduction

As the functional units of any living tissue, cells are considered as the building blocks of life. Their morphology is a large-scale manifestation of various organizing, competing, and highly regulated biological processes^1–5^. Cell morphological analysis^3,6^ thus has long been used in cell biology and physiology for abnormality identification^7,8^, early cancer detection^9,10^, and dynamic change analysis under specific environmental stresses^11,12^. In such investigations, cell biologists usually use cell images provided by optical microscopy to examine cell shape by locating edges, spreading area and so forth, to obtain qualitative and quantitative measurements. Although this approach delivers detailed information about cells’ inner/outer structures, it offers only planar two-dimensional information. However, the pathway to the third dimension, i.e. to the cell thickness imaging, is known to be essential since cell morphology is virtually a result of the mechanical balance^1,13^ between the intracellular components and the outside 3D extracellular microenvironment^14^. A few interferometric imaging techniques such as optical coherence tomography^15,16^, and phase contrast digital holographic microscopy^17,18^ provide access to the in-depth information. However, the axial resolution is limited, generally ranging from a few microns to sub-microns. This is insufficient to study the fine structures of cells of thickness less than 100 nm, for instance the lamella or the leading edge during migration, and thus it hampers progress in morphological phenotyping. Cell roughness is moreover an interesting indicator of the cell’s health state as shown by studies related to diabetes^19^. It can be altered by modifying the membrane ultrastructure^20^, or by disorganizing the cell microstructure, as observed for cancer cells^21,22^. Differential confocal microscopy^23^ and its variation with structured light illumination^24^ are non-interferometric optical profiling techniques showing great potential in the field^25^. Nevertheless an accurate calibration procedure is required and both the accuracy and axial resolution are susceptible^26^ to disturbances of the light source power, ambient lighting, and reflection characteristics of the sample surface. So far, atomic force microscopy (AFM)^27–29^ has been the main tool for 3D single-cell morphology study, by virtue of its nanoscale definition and axial accuracy, despite AFM images are often containing features that are not present on the sample in reality^30,31^ when dealing with biological cells. Such artefacts could be caused by the AFM tip, such as double/triple tip, tip contamination, and especially by the interaction between tip and cells, including collision, contrast reversal and multiple probe-sample contacts, since cells are generally sticky and soft^32,33^. Hence developing a non-contact, label-free and non-invasive method to image the cell’s 3D morphology remains of tremendous significance in the field.

As an elegant combination of light and sound, photoacoustic microscopy is an emerging technique that allows multi-scale imaging of biological samples, ranging from organs to organelles^34,35^. This technique notably takes advantage of the strong absorption of light by haemoglobin to enable high-resolution ultrasound imaging and diagnosis^36–38^. When coupled to an acoustic microscope, single cell images can be obtained. However, the contrast mechanism in photoacoustic microscopy depends primarily on the optical absorption of a cell^39^ or is produced by exogenous chromophores the cell must absorb^40^. As such, photoacoustic microscopy is not suited to label-free quantitative imaging of the single cell 3D morphology.

Using acoustic methods to image biological samples has a noble history in parallel with optical modalities. High-frequency ultrasonics indeed offers an alternative route for high-resolution imaging since the acoustic resolution is similar to the optical resolution when using ultrasound around GHz frequencies^41^. At higher frequencies, ultrasound can potentially provide higher resolution than optical microscopy. For example, scanning acoustic microscopy under cryogenic conditions has shown a capability of ~15 nm in-plane spatial resolution at frequencies as high as 15 GHz for metallic samples in liquid Helium^42^. To date, however, resolution is limited to near sub-micron for biological samples^43^ because the implementation of such a system in ambient conditions is typically limited to 1–2 GHz owing to strong acoustic attenuation in the coupling medium.

The picosecond ultrasonics (PU) technique allows the optical generation and detection of ultrafast acoustic waves, up to THz frequencies^44^, using femtosecond lasers. After decades of being used to study thin solid films, PU has attracted increasing attention in the field of single-cell biology, owing to its two superior features beyond all-optical and non-invasive^45^. One is its direct access to sound velocities in the penetrated volume, thereby opening a new window on cell mechanics at GHz frequencies. After its first demonstration on plant cells in 2008^46^, followed by a variety of mammalian cells, including the human mesenchymal stem, osteoblastic, endothelial, osteosarcoma cells, originating from different tissues^47,48^, Dehoux *et al*. reported 2D optical ultrasonography^49^. It operates in the 10 to 100 GHz range and can be used to map the acoustic impedance and adhesion stiffness in human mesenchymal stem cells and image fine cell structures with thicknesses of tens of nanometers. The other advantage of PU is its high resolution in time and space, particularly along the axial direction. It uses the light controlled emission of coherent acoustic phonons (CAPs) to amplify light scattering and permits the simultaneous time tracking of CAPs in small volumes at depth^50^. By recording the time-resolved Brillouin scattering oscillations, resulting from the propagation of transient acoustic wavefronts through the cell^46^, Danworaphong *et al*.^51^. and Perez-Cota *et al*.^52^ have presented sections of fixed and living cells along the axial direction. The vertical axial definition was determined by the half optical wavelength *λ*/2, ~150 nm and ~300 nm, respectively. In this work, by a combined tracking of both the homogenous propagation of CAPs in cells and of their interaction with the cell outer interfaces, we measure whole cell thicknesses ranging from below *λ*/2 to several *λ* thereby leading to mapping of the full 3D cell morphology remotely.

In the following, the remote thickness measurement is first demonstrated with experiments performed on thin films of cellular-scale thickness, before implementation for biological cell imaging. The 3D remote opto-acoustic mapping of the morphology of an entire osteosarcoma cell is compared to 3D images of the same cell obtained with standard AFM. Special attention is paid to the acoustic resonances and their harmonics, from which the contrast is significantly increased as illustrated with thickness mappings of the nucleus of monocytes and macrophages.

## Results

### CAPs in cell-mimicking films

In this section measurements are performed on cell-mimicking films of controlled thickness. The samples are made of PMMA, spin-coated on the same Ti transducer as used for later biological applications, see Supplementary Information (SI). Two scenarios for the remote opto-acoustic thickness measurement are implemented for thin films of thickness either less than half the optical wavelength *λ* or larger than *λ/*2, with *λ* = *λ*_0_/*n* the probe light wavelength in the sample. The signal analysis and processing for the above-mentioned scenarios are described in Material and Methods where simulated waveforms are considered for illustration.

**Figure 1:**
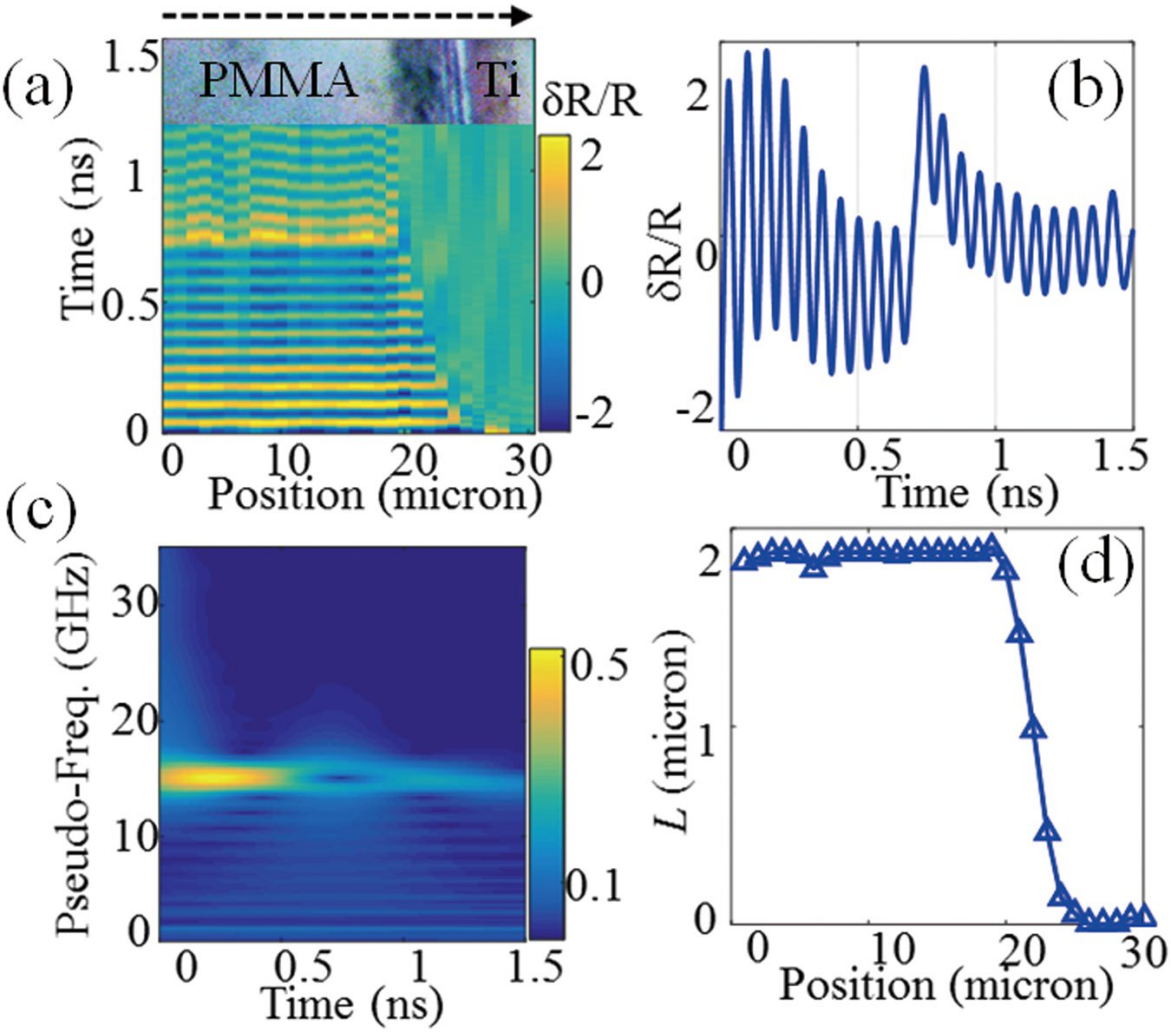
Opto-acoustic profiling of a cell-mimicking film with *L* > *λ*/2. (**a**) Time-resolved waveforms along the scan line that covers both PMMA and bare Ti (inset shows a section of the white light image of the sample surface). (**b**) Measured waveform showing superposition of Brillouin oscillations and step like changes. (**c**) By means of time-frequency analysis, one can determine f_B_ and the time when CAPs reach the top surface τ_0_, thereby leading to recovery of the thickness. (**d**) Following this protocol, the thickness profile of the entire scan line can be obtained.

We first consider a transparent film with a thickness of 1800 ± 30 nm measured with a stylus profilometer. Transient reflectivity signals were experimentally recorded for laser spot positions spaced at 1 µm intervals along a scan line of length 30 µm. The time-resolved waveforms are presented in Fig. 1(a) with a white light image of the sample surface as an inset at the top showing the boundary between thin film and bare titanium. One can clearly identify in this figure the region (0–19 µm) where oscillations of the transient reflectivity are observed up to ~2 ns. The laser spot positions are then over an area covered with PMMA. Measurements at intermediate positions (20–24 µm) reveal the film border where less and less oscillations are detected due to the reduced thickness in the vicinity of the bare Ti region (25–30 µm). For the waveforms collected in the PMMA area, an increase in the reflectivity is observed around 700 ps due to the strong sudden motion of the top free surface (see Materials and Methods). One such experimental waveform is shown in Fig. 1(b) where the signal appears clearly as a superimposition of time-resolved Brillouin oscillations and of stepwise changes, as expected from numerical predictions see Fig. 8(b) in Material and Methods. We performed a Morlet time-frequency analysis, shown in Fig. 1(c), to determine both the Brillouin frequency *f*_B_ = 14.8 GHz and the time when CAPs reach the top surface *t*_0_ = 691 ps. For the probe wavelength (*λ*_0_ = 515 nm) and optical refractive index for PMMA *n* = 1.49 (ref.^53^) we calculated the sound velocity in the PMMA layer of *v* = 2.56 nm/ps with the measured value for *f*_B_. This velocity, together with the measured *t*_0_, yields the film local thickness *L* = 1767 nm. The analysis was repeated for each recorded waveform, leading to reconstruction of the thickness profile along the entire scan line, as shown in Fig. 1(d). The average thickness measured in the area covered with PMMA was calculated at 1779 ± 21 nm, which is in close agreement with the value determined by the stylus profilometer. Note that the standard deviation is of the order of *λ/*20 only. Moreover, the measured averaged thickness value would be an even better fit for the reference value if an optical index *n* = 1.47 was considered for PMMA. This illustrates the capability of the opto-acoustic technique to measure the optical index in situations where the sample thickness is known.

**Figure 2:**
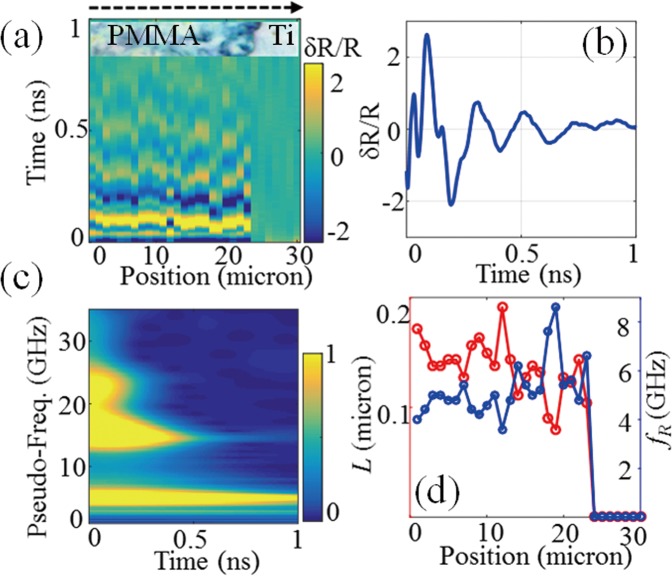
Opto-acoustic profiling of a cell-mimicking film with *L* < *λ*/2. (**a**) Time-resolved waveforms along the scan line that covers both PMMA and bare Ti (inset). (**b**) One typical measurement shows the waveform is dominated by surface motion. (**c**) Its time-frequency spectrum shows a frequency component of long lifetime around 5 GHz attributed to acoustic resonances. (**d**) Using resonance frequencies and sound velocity measured in thicker film, one can reconstruct the thickness profile of the entire scan line.

**Figure 3:**
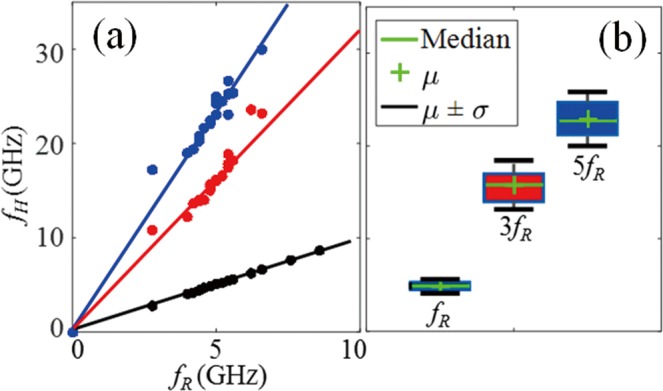
Observation of resonance harmonics for thin PMMA film. (**a**) The correlation between the two high frequency components ${f}_{1}^{H}$, ${f}_{2}^{H}$ and the fundamental frequency (*f*_*R*_) shows a linear relationship. The slopes obtained by linear fittings are 3.2 and 4.7 corresponding to the 2^nd^ and 3^rd^ harmonics, respectively. (**b**) The box-whisker-plots show the measured harmonics are more spread out than the resonance frequencies, which suggests that the frequency of harmonics should provide an enhanced contrast for the imaging of thickness inhomogeneity.

**Figure 4:**
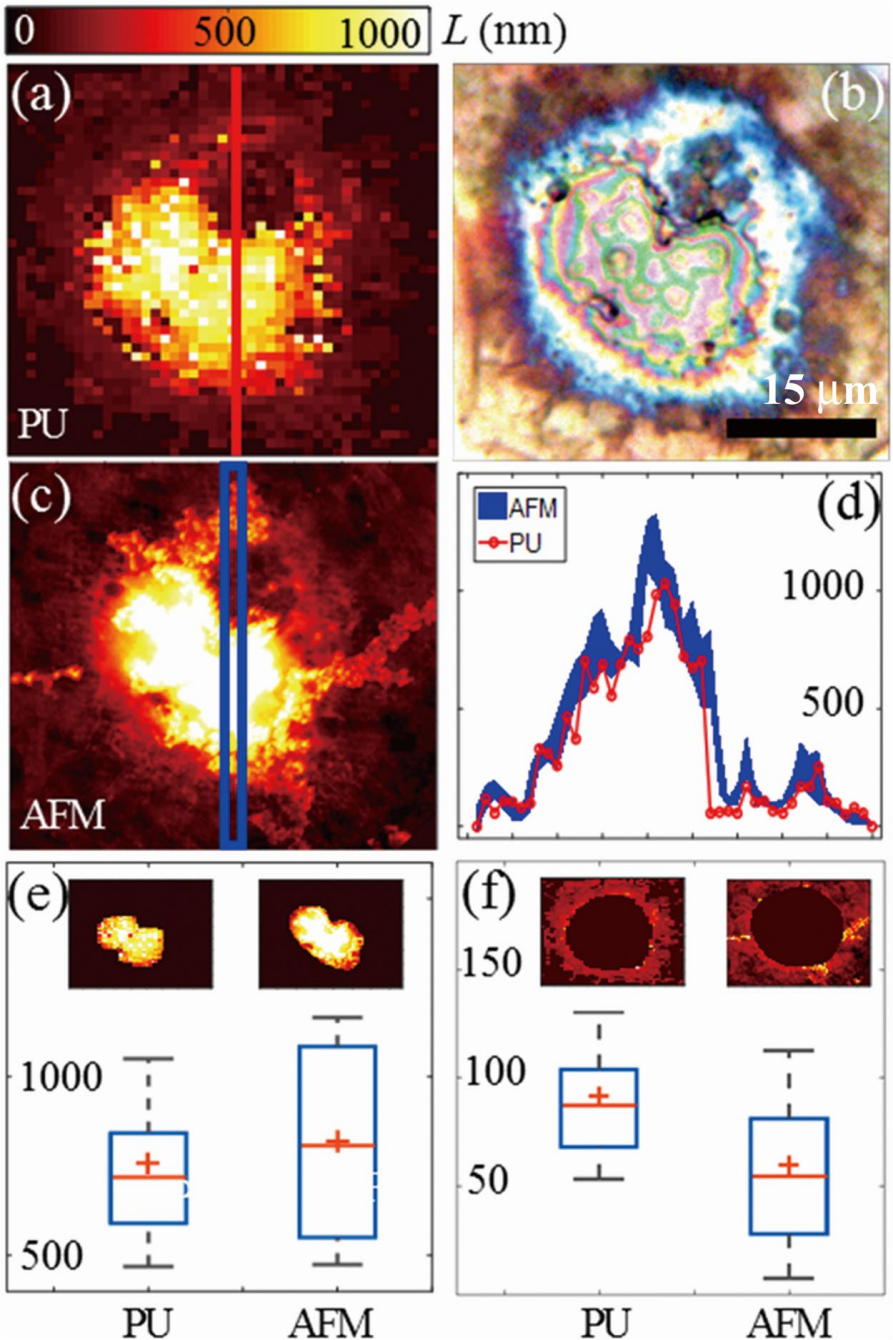
Thickness mapping of an osteosarcoma cell with PU microscopy and AFM. (**a**) Thickness map of an osteosarcoma cell reconstructed by PU microscopy. (**b**) White light top-view image of the osteosarcoma cell. (**c**) Thickness map measurement with AFM. (**d**) Quantitative comparison of the profiles along the selected cell slices shown with rectangle area in (**a**,**c**). Statistics of thicknesses measured in (**e**) the cell nucleus and (**f**) the surrounding cytoplasm of this osteosarcoma cell. Insets in (**e**,**f**) show where the data for nucleus and cytoplasm were situated in the images obtained with PU and AFM.

**Figure 5:**
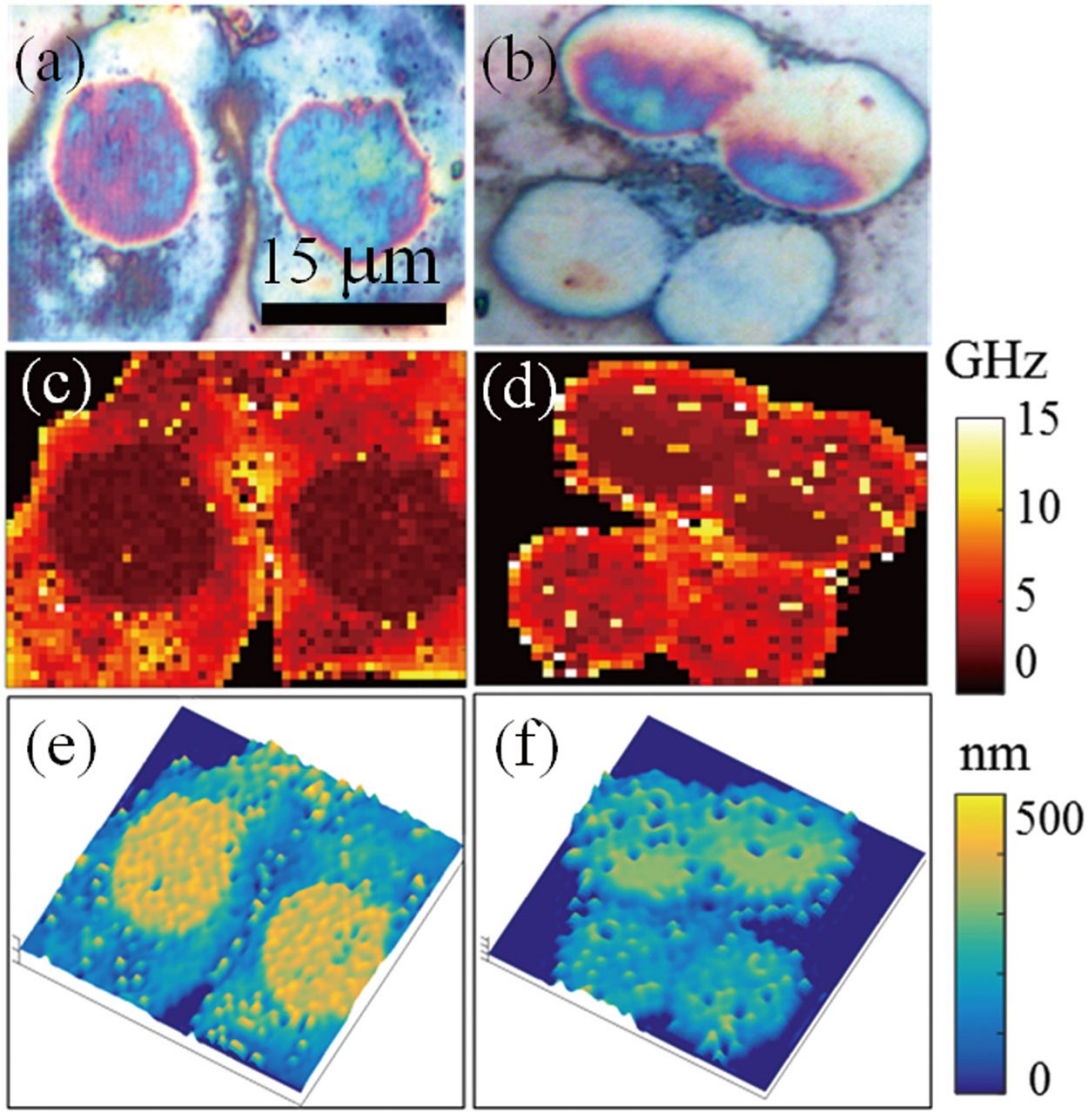
Thickness mapping of macrophages (left) and monocytes (right) with acoustic resonances. (**a**,**b**) White light top-view images. (**c**,**d**) Maps of the acoustic resonances *f*_R_ measured in the recorded PU signals. (**e**,**f**) quantitative 3D cell morphologies.

**Figure 6:**
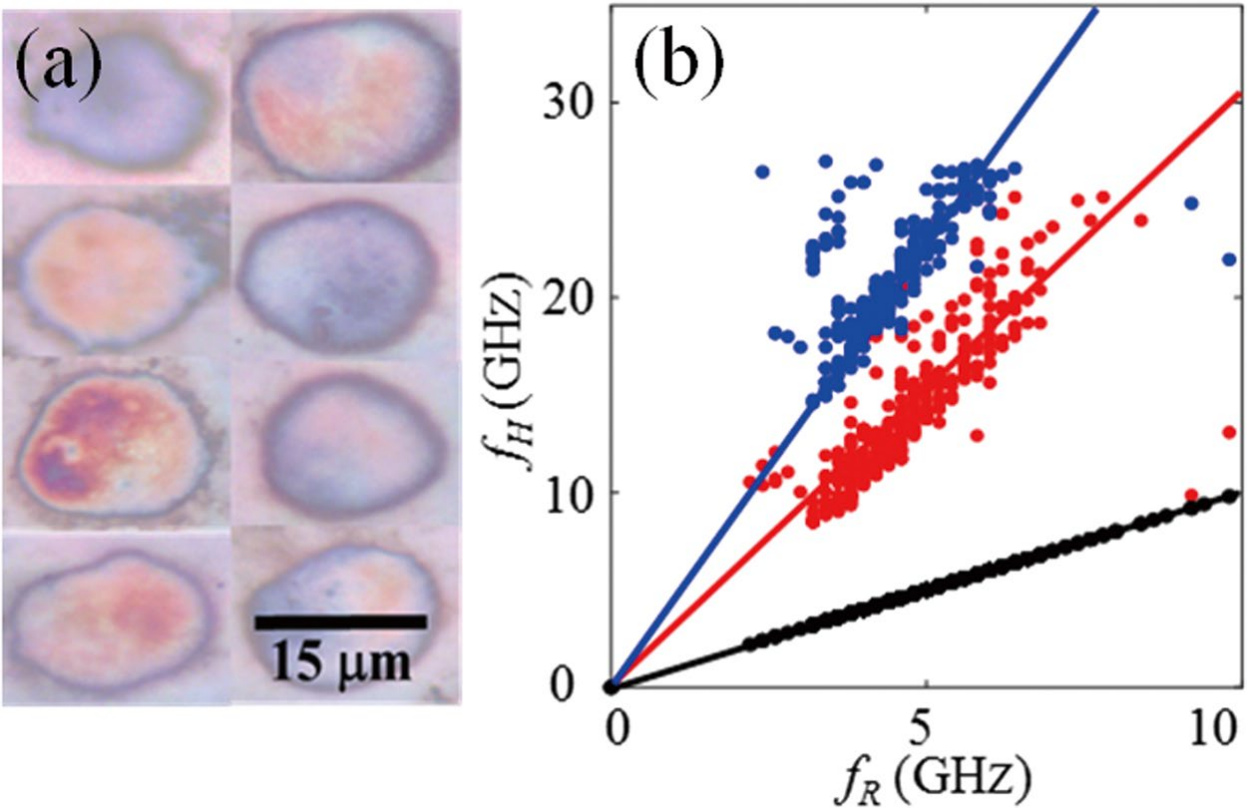
Observation of resonance harmonics for monocytes. For statistical analysis a 4 × 4 scan was performed in the nuclei of 20 cells. (**a**) Top-view white light images of some of these monocytes. (**b**) Linear correlation between the two high frequency components ${f}_{1}^{H}$, ${f}_{2}^{H}$ and the fundamental frequency (*f*_*R*_).

**Figure 7:**
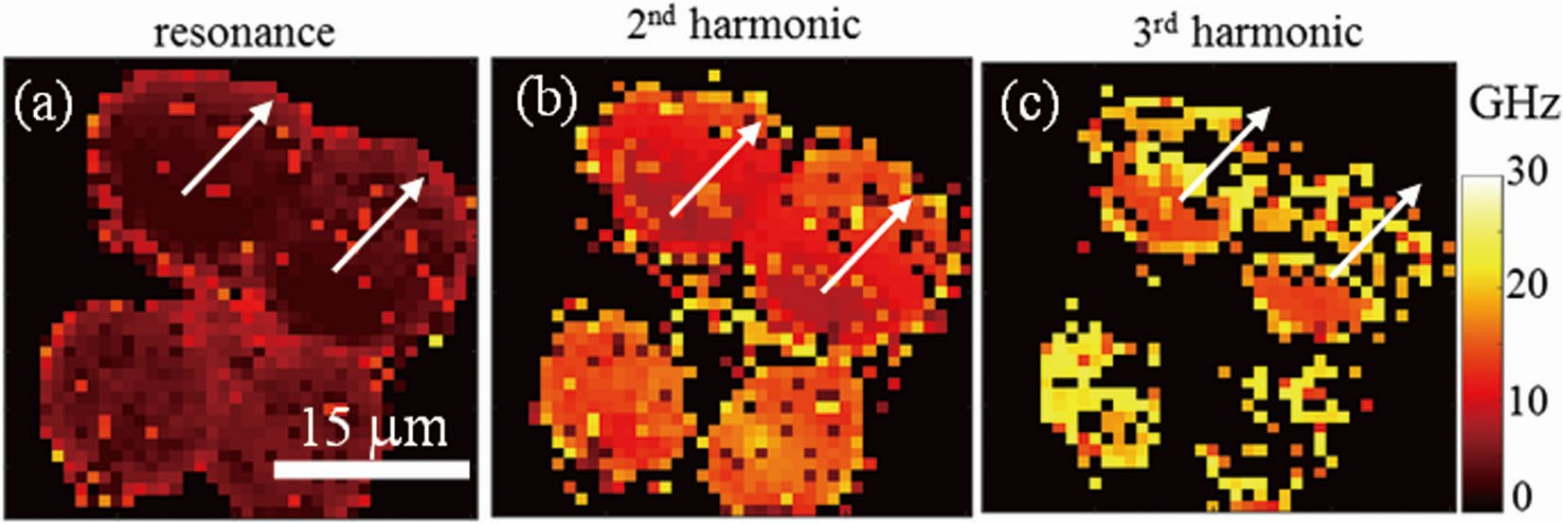
Contrast enhancement provided by harmonics for thickness inhomogeneity imaging. The thickness inhomogeneity is more pronounced in the images obtained with the 2^nd^ harmonics (**b**) and 3^rd^ harmonics (**c**) compared to the image of the first eigenmode frequency (**a**). The white arrows show the migration direction revealed for the two cells at the top of the images.

**Figure 8:**
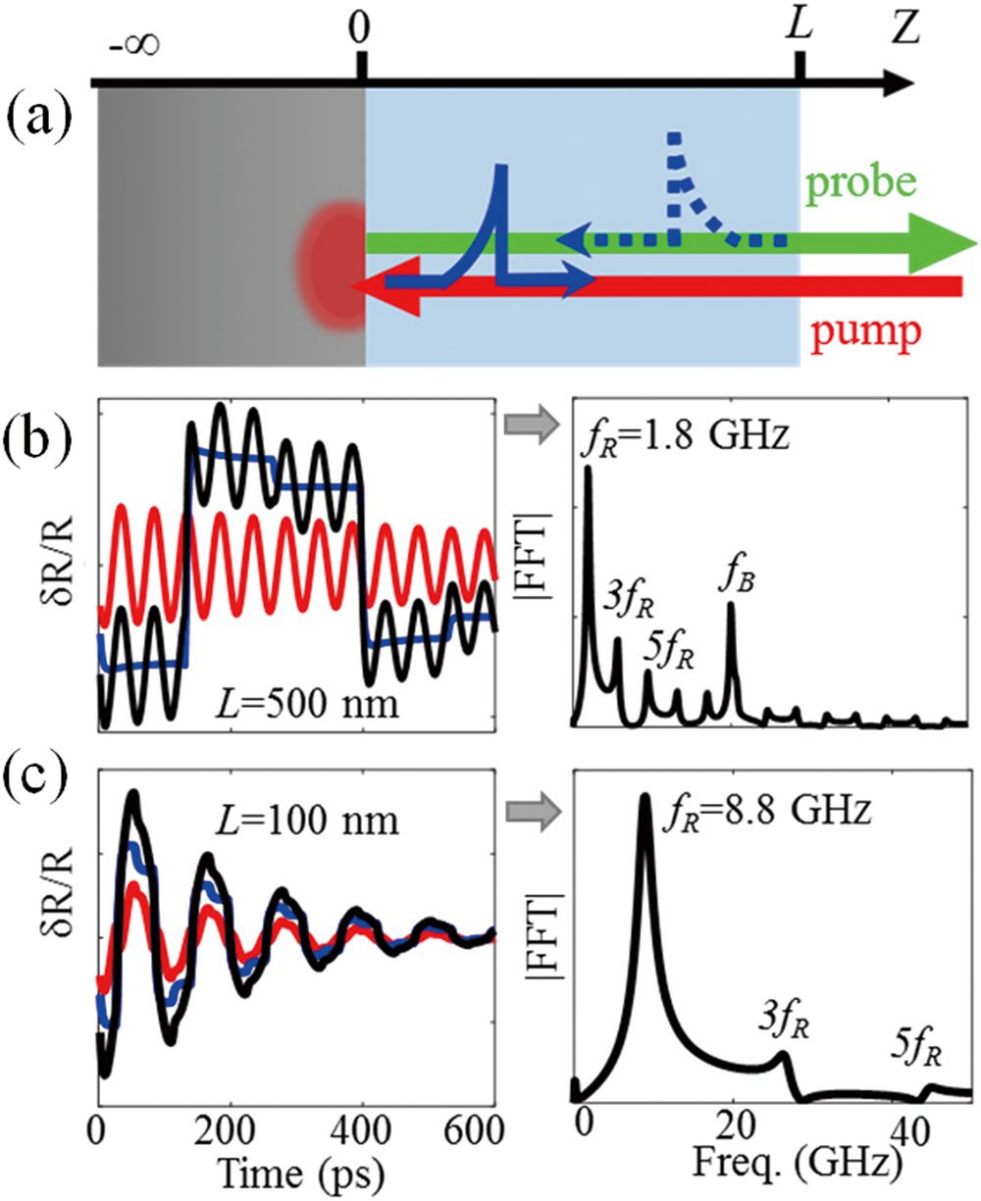
Transient reflectivity of a transparent thin film. (**a**) Schematic of the geometry. (**b**) When film thickness L (500 nm) is larger than one spatial extent of Brillouin oscillations *λ*_*B*_ (190 nm) the reflectivity variation (black) is a superposition of the Brillouin oscillations (red) and step-like motion (blue), as also illustrated with the FFT spectrum (right panel). (**c**) When L (100 nm) is less than *λ*_*B*_, the waveform (black) is dominated by the step motions (blue) while the Brillouin oscillations (red) are only fragmentally discernible at the very beginning, as confirmed by the FFT spectrum (right panel) which reveals the first acoustic eigenmode and the harmonics only.

We now illustrate the second scenario, where the film thickness is less than the spatial extent of one wavelength *λ*_*B*_ of the CAP that gives rise to the Brillouin acousto-optic interaction. Figure 2(a) presents the waveforms recorded along a scan line across a thin PMMA film of 150 nm in thickness. The region covered with film and the bare Ti region can still be discriminated from the map of waveforms, and correspond well with the optical microscopic picture (inset). As expected, short-time oscillations tend to dominate the signals in this case. Figure 2(b) shows one representative waveform in which Brillouin oscillations are only present fragmentally at the very beginning and can barely be identified. This observation is confirmed in the time-frequency spectrum (SI) shown in Fig. 2(c) where a frequency component with a lifetime up to 1 ns is observed around 5 GHz, as a result of the step motions of the interfaces at frequency *f*_R_. Keeping in mind that this frequency is that of an acoustic quarter wave plate and using the sound velocity measured in the thicker film, one gets the local thin film thickness of *L* = *v*/4*f*_*R*_. Using the resonance frequency profile shown on the right axis in Fig. 2(d) we measured the entire thickness profile of the thin film, as indicated on the left axis of the figure. The average thickness is calculated at 145 ± 27 nm, which is again consistent with the reference value, 150 ± 10 nm, measured using the stylus profilometer.

In addition to *f*_R_ it is interesting to observe, in the time-frequency spectrum, the presence of two other components at higher frequencies. These components are at frequencies ${f}_{1}^{H}$ = 15 GHz and ${f}_{2}^{H}$ = 23 GHz about 3 times and 5 times that of the first eigenmode *f*_R_. This is clearly experimental evidence of the detection of the 2^nd^ and 3^rd^ harmonics of *f*_R_, (Materials and Methods). Those harmonics were determined for each signal measured along the scan line in the thin PMMA film, and the correlation between the two high frequency components and the resonance frequency is plotted in Fig. 3(a). A linear relationship is clearly observed for both frequencies but with different slopes, 3.2 and 4.7 respectively, confirming the measurement of 2^nd^ and 3^rd^ harmonics. The reason for the two ratios being not exactly at 3 and 5 can be attributed to the reduced frequency resolution when using the Morlet wavelet to perform time-frequency analysis, as addressed in the SI. It is also worthy of note that the measured harmonics are more dispersed than the fundamental frequencies, as it can be seen in the box-whisker-plots in Fig. 3(b). In such plots, the top/bottom horizontal lines, determined by standard deviations (±1σ), 0.7 GHz, 2.6 GHz, 2.8 GHz from left to right, represent the spreading of the measured frequencies for *f*_*R*_, 3*f*_*R*_ and *5f*_*R*_, respectively. This observation illustrates that the harmonic frequencies are more sensitive to thickness variations than the first eigenmode frequency (Materials and Methods), suggesting that harmonics can be used to enhance the contrast of thickness inhomogeneity imaging. In the box-whisker-plots, the cross inside the box represents the mean frequency, which is quite close to the median frequency (line inside the box), verifying that the reconstructed data is not skewed.

### 3D morphology of an entire osteosarcoma cell

In the previous section we demonstrated two complementary scenarios, both based on the PU tracking of CAPs, to measure thin film thickness. This suggests an all-optical and non-invasive approach for quantitative imaging of the morphology of thin transparent samples with thickness ranging from a few optical *λ* down to sub *λ*/2. In this section we explore the possibility of mapping single cell 3D morphology remotely, which is of significant importance to aid in the understanding of complex processes involved in cell biology and physiology.

To illustrate the ability of the PU microscope to map the surface of highly structured cells we started with osteosarcoma cells, since it is the most common histological form of primary bone cancer and as such the surface of these cells shows strong roughness^21,22^. They were cultured and fixed on a titanium plate (see SI) the surface of which was scanned in a raster manner within a 45 × 45 µm^2^ square with 1 µm steps. The diameter of the optical probe spot sets the acoustic lateral resolution, comparable to conventional diffraction-limited optical imaging techniques. With the present laser wavelength and objective lens magnification, the diameter of the probe spot is ≈1 µm. We performed PU measurements (a few seconds per pixel) to map an entire osteosarcoma cell and produced acoustic movies with a frame rate of 1 image/ps (see Online Movie 1).

The two routes for thickness measurement were combined, to reconstruct the whole thickness map presented in Fig. 4(a). For this, a constant optical index value given in Table S1 was assumed in the thick parts where the sound velocity was measured at each pixel, and the average value of these velocities was considered to calculate the thicknesses of the thin parts of the cell. The effect of the cell mechanic and optical inhomogeneity, at either the nano or micron scale, on the thickness measurement accuracy is discussed in SI. The thicker part in the centre and thinner part in the surrounding area represent the cell nucleus and cell cytoplasm, respectively. Their contours correspond well with the microscopic image of the cell, Fig. 4(b), taken with the built-in white light reflection microscope. The images also show the cell is binucleated and has a large cytoplasmic vacuole.

For quantitative 3D comparison, we scanned the surface of this cell by means of an AFM since such a device is, so far, the gold-standard tool to map 3D morphology for single-cells. After processing raw data provided by the AFM (SI, Fig. S5) we obtain the cell thickness map shown in Fig. 4(c). The two techniques provide very similar thickness distributions, as even more clearly illustrated with the 3D representations in Fig. S6 in SI, despite AFM delivers more details of cell morphology owing to higher lateral definition, 0.12 µm compared to 1.0 µm for PU microscopy. It should be noticed that the cell thickness map obtained with PU appears pixelated in Fig. 4(a) because the step of the scanning was set equal to the current lateral resolution of the PU microscopy, 1 µm. However the pixilation can be suppressed by using higher magnification objective lenses and/or by using finer scan steps as in many other pump-probe laser scanning microscopy techniques^54^.

In order to compare the two measurements quantitatively, we plotted in Fig. 4(d) the profiles along 45 µm of a vertical cell slice shown with red and blue lines in Fig. 4c, respectively. The width of this slice, 1 µm, is that of one pixel in the image obtained with PU microscopy. To account for the different lateral definition each value in the vertical profile obtained by AFM is an average over a square of 9 × 9 pixels in the AFM image. The thickness of the plotted profile is representative of dispersion of the AFM data within this square. Comparison of these profiles shows that the two measurements are very consistent both in the thin, *L* < *λ*/2, and thick parts of the cell. Some small shift in the thick part of the cell could be attributed to two main reasons. First, the AFM measures the height profile of the sample surface whereas PU measurements reveal the cell thickness, regardless of the titanium-cell interface profile. Some local differences, showing lower value for PU data, can thus be caused by bumps on the titanium surface. Second, the local disagreement may be attributed to an under estimation, in some parts of the cell, of the sound velocity, as measured with the Brillouin frequency, due to an over estimation of the cell optical index. This was illustrated by preliminary measurements we performed on the thick (1.8 µm) PMMA film.

A further comparison was carried out by means of statistical analysis, shown in Fig. 4(e) and (f), of the thickness data measured with the two imaging techniques in nucleus and cytoplasm of the osteosarcoma cell, respectively. The mean thickness obtained by the PU microscopy in the nucleus and surrounding cytoplasm is 760 nm and 92 nm, respectively, compared to 820 nm and 61 nm measured with AFM. Note that the mean thickness measured in cytoplasm is around *λ*/4. The good agreement confirms the reliability of PU microscopy as a means of cell thickness-mapping for studying cell 3D morphology. Furthermore, the standard deviations (STD) of thickness values obtained by PU microscopy in both regions are also close to those obtained by AFM, 291 nm and 39 nm compared to 346 nm and 53 nm in nucleus and cytoplasm, respectively. Such STD values are representative for osteosarcoma cell roughness, which has been proved to be an important morphological phenotype^19,20^. We thus demonstrate the accuracy of the reported method for cell thickness measurements and the ability to remotely quantify cell roughness of the order of *λ*/10. These results also illustrate that the method is suitable for studies focusing on the thickness of a limited cell region-of-interest, for instance a part of the nucleus or of the lamelipodium, as it is often performed for AFM-based morphology analysis.

### 3D morphology of macrophages and monocytes

When the maximum cell thickness is comparable to or less than the wavelength of CAPs that give rise to the transient Brillouin interaction, *λ*/2, the signature of this interaction is not detectable for any part of the cell (Materials and Methods). In this section we demonstrate that the complete 3D morphology of thin cells can however be imaged with PU microscopy, based on the analysis of the CAP resonances and of their harmonics.

Macrophages and their precursors, monocytes, were grown on titanium transducers (see SI). For each sample we scanned in a raster fashion a square of size 45 × 45 µm^2^ including either 2 or 4 of these cells, as shown with the white light images in Fig. 5(a,b). The transient reflectivity changes at a ps time scale are provided in Online Movie 2 and Movie 3 for macrophages and monocytes, respectively. For each cell type, acoustic resonances at frequencies *f*_R_ were detected for signals measured in the cell nucleus and also in the surrounding cytoplasm. The frequency maps are plotted in Fig. 5(c,d) for macrophages and monocytes, respectively. These resonance frequencies were converted into thicknesses assuming sound velocity of 3.5 nm/ps, as measured with PU in several cell types^47^. Figure 5(e,f) present the 3D morphology we obtained for macrophages and monocytes, respectively.

Macrophages show a typical elongated oval shape, due to cell spreading on the Ti surface, where one can easily recognize the cell nucleus and cytoplasm. We selected the data for each organelle and calculated thicknesses of 380 ± 41 nm for macrophage nuclei. These cells are about twice as thin as osteosarcoma cells and, not surprisingly, the nucleus surface roughness we estimated from the standard deviation of PU measurements is less, by a factor of 7.5. The data for the macrophages cytoplasm gave a thickness of 164 ± 78 nm. In Fig. 5(c) some regions of thickness as low as 50 nm can be well identified near the cell boundary, which is the leading edge of the cells undergoing spreading.

Compared with the macrophages, the monocytes exhibit a more symmetrical round shape, and tend to be smaller and thinner, as can be seen in Fig. 5(f), with a mean thickness of 206 ± 75 nm. It is noteworthy that the two monocytes located at the top have a more dispersed thickness distribution, 226 ± 79 nm compared to the two at the bottom, 192 ± 42 nm, due to the presence of a nucleus thickness gradient as a result of cell migration. This illustrates that the reported approach can be used to map the thickness inhomogeneity that is generally involved in cell migration and in other cell processes as well.

In addition to *f*_R_ we observed also the presence of other components at higher frequencies in the time-frequency spectra of the waveforms collected for monocytes. In order to confirm that these components are indeed harmonics of the first eigenmode in cells, a statistical study was carried out from measurements performed with 20 individual cells. For each cell, we recorded the transient opto-acoustic response along a 4 × 4 scan of the nucleus. Figure 6(a) shows top-view bright-field images of several cells selected for inspection. The correlation between the higher frequencies and the resonance frequencies is illustrated in Fig. 6(b). We clearly identified two linear-correlated distributions with slopes of 3.1 and 4.6 corresponding to 2^nd^ and 3^rd^ harmonics, respectively, as was observed in Fig. 3(a) for measurements performed on the thin PMMA film. This confirms the detection of harmonics in monocytes, and also shows that the reported method can be used to study 3D cell morphology in two ways, either by complete mapping or using statistics over limited measurements performed in several cells.

Since the physical origin of these higher frequencies is now confirmed we map in Fig. 7(b,c) the 2^nd^ and 3^rd^ harmonic frequencies we measured for the monocyte cells already imaged with the first eigenmode frequency only, Fig. 7(a). Comparison of these images shows the thickness inhomogeneity is more pronounced in the images provided by harmonics. As expected, this contrast enhancement benefits from the inherent gain factor of 3 and 5 from the 2^nd^ and 3^rd^ harmonics, respectively. The images reveal the two cells at the top of the images were undergoing migration in the direction of their nucleus thickness gradient, shown with white arrows in Fig. 7.

## Discussion

We used optically generated and detected CAPs at GHz frequencies to perform remote 3D imaging of cell morphology. This modality was inserted on a regular upright microscope, making it handy to cooperate with the built-in bright field and fluorescence microscopies, so that complementary images could be obtained simultaneously. The proposed approach was first applied to thin cell-mimetic films with cell-scale thickness. The analysis relies on the time-resolved Brillouin spectroscopy of the acousto-optic interaction along the cell depth, and on CAP resonance spectroscopy. The implementation was supported with the 1D modelling of the measured reflectivity changes. We successfully measured film thicknesses less than *λ*/2 and reported an accuracy better than *λ*/20.

We first applied the remote method to an osteosarcoma cell, the roughness of which is known as biologically relevant. The entire 3D morphology of the same cell was also obtained using AFM as a reference mean for contact thickness measurement. The good agreements of the measured profiles and mean thicknesses confirm accuracy of the remote technique for measuring cell thicknesses as small as *λ*/7. In addition, we show that the in-plane definition of 1 µm, as limited by optical diffraction, is convenient for performing relevant roughness analysis for cancer cell. A sensitivity to cell roughness of the order of *λ*/10 is demonstrated.

We next paid special attention to the first acoustic eigenmodes in cells. By detecting such acoustic resonances, we obtained the thickness map of macrophages and of their precursors, monocytes. As expected, the results show that macrophages are generally thicker than monocytes. In addition, we demonstrated the detection of 2^nd^ and 3^rd^ harmonics in monocytes. Taking advantage of their higher sensitivity to cell thickness, mapping the harmonics’ frequencies revealed the inhomogeneity of the cell nuclei. The nucleus thickness gradient we measured is consistent with that of a migrating cell.

Finally, the possibility of using CAPs to image cell morphology in 3D was proven, and potential applications for biologically relevant studies were illustrated with measurements of cell roughness and of nucleus deformation during migration. We have shown that 3D PU morphology is a new imaging modality that can be implemented onto a regular upright light microscope, and that could be used as an alternative method to AFM for such cell studies. These results open the path for wide applications in biology and medicine where remote measurement of the cell 3D-morphology is required.

## Material and Methods

### CAPs and thin films thickness retrieval

To introduce the physical mechanism in play for thickness retrieval from the picosecond opto-acoustic waveforms, we consider a sample geometry consisting of a thin transparent film of thickness *L* adhering to a semi-infinite absorbing substrate, as illustrated in Fig. 8(a). Upon absorption of a sub-picosecond pump laser pulse by the metal opto-acoustic transducer, a sudden temperature rise occurs in the metal in the vicinity of the absorption zone. The ensuing transient thermal expansion launches a strain pulse, which propagates in the thin film and modifies the optical reflectivity of the sample to the probe laser pulses.

An analytical simulation model was developed to illustrate the reflectivity changes. It accounts for the photo-thermal and thermo-acoustic transduction and integrates the elasto-optic interaction along the whole structure^55^. We consider a sample made of a titanium half-space covered with a layer representative of a typical cell, with physical properties given in Table S1 (Supplementary Information, SI). In this geometry, the measured relative reflectivity changes *δR*(*t*)/*R* are typically featured by the so-called time-resolved Brillouin oscillations^56^, arising from the acousto-optic interaction. The frequency of the oscillations is *f*_*B*_ = 2*nv*/*λ*_0_ in the case of normal incidence, with *n* being the optical refractive index of the film material, *v* the longitudinal sound velocity, ~few nm/ps, and *λ*_0_ the light wavelength of the probe laser in vacuum, 515 nm in this work. In addition, owing to the finite thickness of the transparent film, acoustic nano-pulses propagate back and forth through the film and cause periodic interface displacements at both the *z* = 0 and *z* = *L* interfaces. These are revealed by equally spaced step-like changes in the reflectivity *δR*(*t*)/*R* of the sample, with the transparent film acting as a Fabry-Pérot cavity^57,58^.

We first scrutinize the case where the film thickness is larger than the wavelength *λ*_*B*_ = *λ*/2 of the CAP that gives rise to the Brillouin acousto-optic interaction, with *λ* = *λ*_0_/*n* the probe light wavelength in the sample. Since with the material and probe wavelength involved we obtain *λ*_*B*_ = 190 nm, in Fig. 8(b) we plot the transient relative reflectivity for a thin film of thickness *L* = 500 nm. At *t* = *0*, the launched strain pulse immediately causes a weak negative step jump at the *z* = 0 interface. Since detection of the interface displacement relies on the interferences between probe reflection at both interfaces, the sign of the reflectivity jump depends on the thickness, relative to the probe optical wavelength^59,60^. When reaching the mechanically-free surface, *z* = *L*, the compressive strain pulse is converted into a pure tensile strain and leads to a second but more abrupt jump at *t*_0_ = *L*/*v*, ~140 ps. Since the lifetime of CAPs is long compared to *z* = *L*, multiple reflections of the acoustic wave are observed at integer multiples of *t*_0_. However, jumps occurring at the layer/substrate interface at even-multiples of *t*_0_ are not very pronounced, owing to the impedance of the considered materials. As a result, the step motions are dominated by jumps at odd-multiples of 4*L/v*, giving rise to a quasi-periodic square signal with a period of 4*L*/*v*. Overlapped on this step-like signal are the Brillouin oscillations resulting from the interaction of the probe light with the acoustic nano-pulses in the transparent layer. They are more clearly represented in the Fourier spectrum of the waveforms, as shown in Fig. 8(b). In addition to *f*_*B*_ = 20 GHz several other frequency components are observed. The spike of large amplitude at *f*_R_ is followed by equally-spaced peaks of decreasing amplitude. Assuming purely linear mechanic behaviour these peaks result of the interferometric detection of the periodic motion of the free surface of the film. Their decrease with increasing frequency depends primarily on the acoustic leakage due to partial reflection at the film-substrate interface, and on the acoustic absorption in the film. On that account the changes of the peak amplitudes and width with frequency would give interesting information on phonon relaxation and on the medium structure at the nanoscale. Assessing this information for cells is however outside the scope of this work. We instead focus on the peak frequencies that are directly related to the transparent layer thickness. Owing to the acoustic mismatches at the interfaces the film is comparable to an acoustic quarter wave plate with eighenmode frequencies *f*_*n*_ = (1 + 2*n*)*v*/4*L*. The spike of large amplitude at *f*_R_ = 1.8 GHz in Fig. 8(b) is thus attributed to the first eigenmode of a quarter wave plate. The peaks at frequencies 5.4 GHz, 9.0 GHz, odd-multiples of *f*_R_, are the signatures of eigenmodes of higher order denoted as 2^nd^ and 3^rd^ harmonics in the following, respectively. The higher sensitivity of the harmonic frequencies to the film thickness *L* will be illustrated experimentally in the following of the paper.

We next calculate the reflectivity changes *δR*(*t*)/*R* assuming the film thickness is less than the spatial extent of the CAPs giving rise to Brillouin detection, *λ*_*B*_ = *λ*/2. The transient time response calculated for *L* = 100 nm is displayed in Fig. 8(c). For such a film thickness the signal is mainly dominated by the step motions (blue) while Brillouin oscillations, appearing fragmentally at the very beginning (red), can hardly be identified. This is underlined by the FFT spectrum (bottom panel) where *f*_*B*_ is absent, while the first eigenmode at *f*_*R*_ = 8.8 GHz and the harmonics are observed.

The method for cell thickness imaging consists in first measuring the CAPs velocity from the Brillouin signal frequency in the thick parts of the cell, then calculating the thickness from either time or frequency analysis of the step like signals, as described in SI. Its application to measuring solid films^59,61,62^, and fixed cells^55^ thickness at a single point has been reported. In section II, the remote imaging of cell thicknesses ranging from a few microns to sub-100 nm is demonstrated and the resulting 3D cell morphology is compared with that achieved with AFM for the same cell. An improved contrast is obtained when eigenmodes of higher orders are considered.

### Experimental setup for tracking CAPs

**Figure 9:**
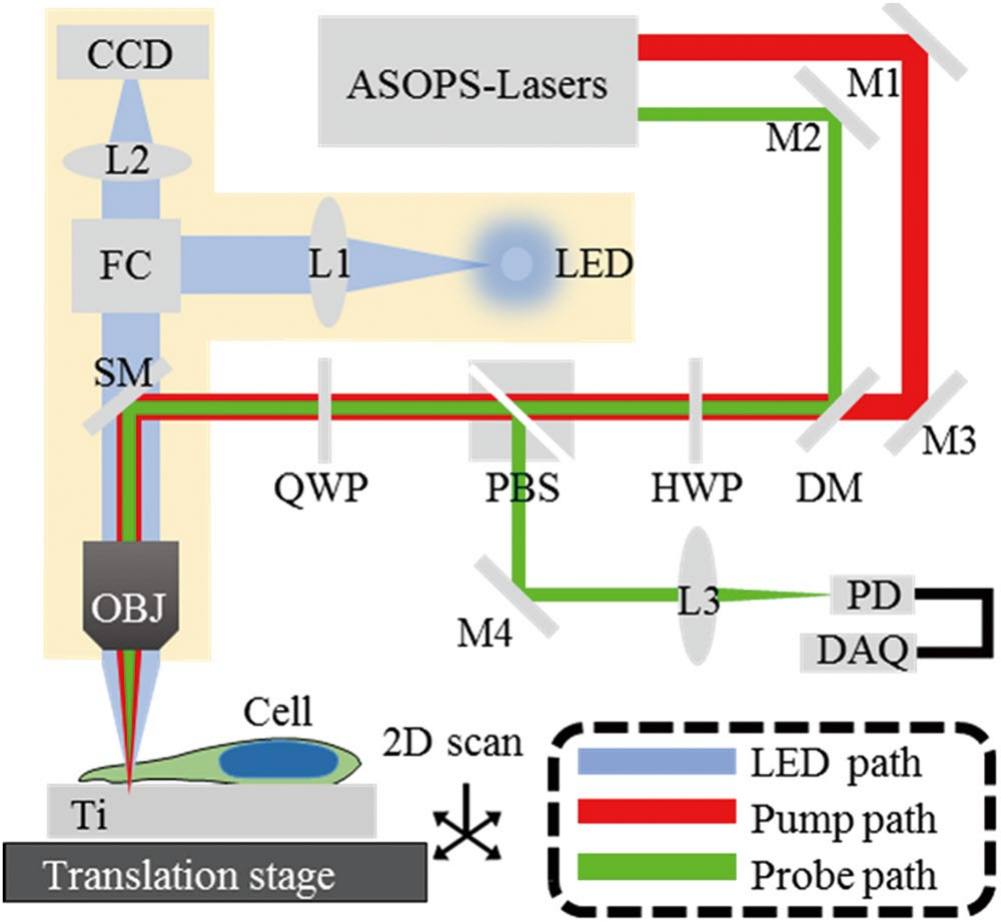
Schematic diagram of the experimental setup. Two lasers with slightly different repetition rates are used to achieve generation and detection of CAPs in cells. Such asynchronous optical sampling (ASOPS) apparatus is hosted in a commercial optical reflection microscope (yellow shaded area), hence allowing convenient access to reflected bright-field observation and/or the reflected fluorescence observation by simply switching the filter-cubes (FC). L, lens; M, mirror; DM, dichroic mirror; SM, switchable mirror; OBJ, objective lens; WP, wave-plate; PBS, polarized beam splitter; FC, filter cube; PD, photodetector; DAQ, data acquisition unit. LED, light emitting diode. A translation stage allows raster scanning of the sample surface.

The all-optical tracking of CAPs was performed using the setup shown in Fig. 9. A dual-oscillator delivers two trains of laser pulses of duration 400 fs, one serving as the pump (1030 nm) and the other as the probe (515 nm). The two lasers are operated at repetition rates of approximately *f*_0_ = 48 MHz, with a slight shift, Δ*f*, to enable asynchronous optical sampling (ASOPS)^60^. The two beams are coaxially aligned and focused by an objective lens, x 50, through the sample onto the surface of a Ti opto-acoustic transducer. The sample holder is mounted on a translation stage, thus allowing raster scanning of the sample with high precision. At the sample-Ti interface, each pump pulse launches a longitudinal strain pulse, which alters the intensity of the reflected probe light during propagation. A notable feature of this setup is its high compatibility with other commonly used microscopic imaging means. The main apparatus involved in the PU detection is fitted onto a commercial upright light microscope, as shown in the shaded area in Fig. 9. By translating the switchable mirrors that are located between the objective nosepiece and the filter cube, it is a simple matter to switch the function of the setup between PU study and reflected-field microscopy. Thus the device not only allows precise alignment of the laser beams onto a targeted cell sample, but also gives convenient access for inspection of other microscopic features of the cell. A more detailed description of the apparatus and implementation of the set-up is given in SI.

## Supplementary information

Supplementary information to Remote imaging of single cell 3D morphology - Rev

Movie 1- Osteosarcoma

Movie 2- Macrophages

Movie 3- Monocytes
