# Supplementary material for: Remote imaging of single cell 3D morphology with ultrafast coherent phonons and their resonance harmonics
Source: Sci Rep. 2019 Apr 23;9:6409. doi: 10.1038/s41598-019-42718-5 (PMC6478725; doi:10.1038/s41598-019-42718-5)
Supplement: Supplementary file 1 — Supplementary information to Remote imaging of single cell 3D morphology - Rev [file 41598_2019_42718_MOESM1_ESM.docx]

**Supplementary information for the article:**

**“Remote imaging of single cell 3D morphology with ultrafast coherent phonons and their resonance harmonics”**

Liwang Liu^1^, Alexis Viel^1^, Guillaume Le Saux^2^, Laurent Plawinski^2^, Giovanna Muggiolu^3^, Philippe Barberet^3^, Marco Pereira^4^, Cédric Ayela^4^, Hervé Seznec^3^, Marie-Christine Durrieu^2^, Jean-Marc Olive^1^, Bertrand Audoin^1^

*^1^University of Bordeaux, CNRS UMR 5295, I2M, F-33400 Talence, France.*

*^2^University of Bordeaux, CNRS UMR 5248, Bordeaux INP, CBMN, F-33600 Pessac, France.*

*^3^University of Bordeaux, CNRS UMR 5797, CENBG, F-33170 Gradignan, France.*

*^4^University of Bordeaux, CNRS UMR 5218, IMS, F-33400 Talence, France.*

SUPPLEMENTARY INFORMATION

The following is the supporting information related to the paper entitled “Remote mapping of single cell 3D morphology with ultrafast coherent phonons and their resonance harmonics”.

**Physical quantities used for simulations**

The effect of film thickness on the reflectivity changes is illustrated with simulation results in Section II.1, in order to discuss the physical mechanism in play and to introduce the reported thickness measurement method. For the simulations, literature values were used for the optical and acoustic parameters of Ti and cells as summarized in Table S1.

|  | Ti (ref. 1) | Cell (ref. 2) |
| --- | --- | --- |
| Optical absorption coefficient (m^-1^) | 5.2×10^7^ | 0 |
| Optical refractive index | 2.42 | 1.35 |
| Density (kg.m^-3^) | 4500 | 1150 |
| Longitudinal sound velocity (nm.ps^-1^) | 5.9 | 3.6 |
| Longitudinal elastic modulus (GPa) | 155 | 14.9 |
| Longitudinal loss modulus (GPa) | 0 | 1.3 |

*Table S1: Optical and acoustic parameters involved in the simulations.*

**Time-frequency analysis**

In order to retrieve the film thickness from reflectivity changes,* δR*(*t*)*/R,* one has to determine the sound velocity *v* of CAPs in the film and the time when CAPs reach the top surface of the sample for the first time, namely *τ*_0_. In this work the two quantities were identified with a signal processing method based on a time-frequency analysis. We used a wavelet transform with a mother Morlet wavelet, simply expressed as the product of a sine wave and a Gaussian envelope, Eq. (S1).

$W_{m}(t)=\cos(2\pi ft)\exp[-(t/s)^{2}/2]$ (S1)


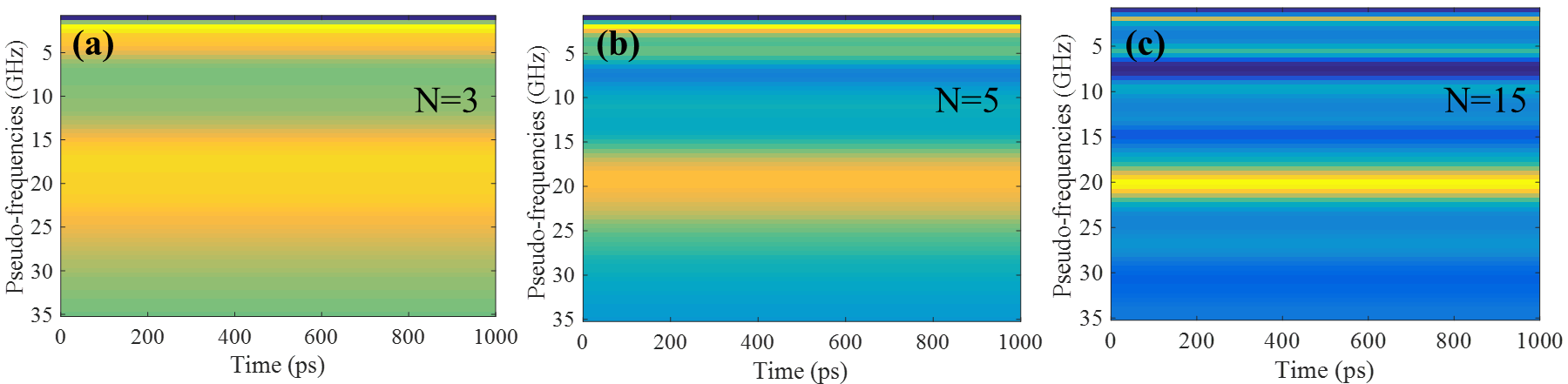


***Figure S1*** *Time-frequency spectrum obtained with Morlet wavelets. The time-frequency spectrum of the simulated waveform shown in Fig. 1(b) was calculated using the mother Morlet wavelets with different N values, 3, 5, and 15, respectively from left to right. The larger N the better frequency resolution is observed as expected.*

In Eq. (S1), *s* is defined as *s*=*N*/($2\pi f$), with *N* the number of wavelet cycles within the wavelet envelope. It controls the width of the mother wavelet frequency. Attention must be paid to the selection of this parameter having in mind the desired trade-off between temporal and frequency precision. By giving *N* a large value one can expect high frequency precision at the cost of time precision, and *vice versa*. Hence the configuration should be adapted according to the application. By convolving the measured *δR*(*t*)*/R* with Morlet wavelets at given central frequencies, also called pseudo-frequencies, one can obtain the time-frequency spectrum of the measured waveform, from which it is possible to identify time distribution and/or time-evolution of frequency components of interest. Fig. S1 presents the time-frequency spectrum of the simulated waveform shown in Fig. 1(b), calculated at different *N* values, 3, 5, 15 respectively from left to right. Fig. S1 shows that as *N* increases the frequency resolution gets finer, approaching the frequency resolution of the FFT spectrum (50 MHz in this work). The frequency spectrum obtained by the Morlet transform at different *N* values, i.e. vertical lines in Fig. S1, is presented in the right axis in Fig. 2(a) and compared with the FFT spectrum, shown in the left axis, of the unprocessed simulated signal. Apparently, it is only when *N*=15, that one can expect to determine the frequency components of interest precisely. However, time accuracy is lost, as illustrated in Fig. S2(b) where the convolution of the simulated reflectivity change with a wavelet of central frequency *f*_B_ is plotted for different *N* values accordingly. The resulting signals feature a series of successive peaks. Their time-width broadens as *N* increases. A narrower width will lead to a more accurate detection of the time location of such peaks, thus to a more reliable measurement of *τ*_0_. The vertical line in Fig. S2(b) indicates the targeted time location for the first abrupt change, *τ*_0_, calculated with the known velocity and thickness. It can be determined when *N*=15, with an offset of ~3 ps that corresponds to an error of~10 nm only. In this work, in order to measure both *f*_B_ and *τ*_0_ precisely, we therefore performed the wavelet transform twice for each waveform. In the first one a mother Morlet wavelet of high frequency resolution (*N*=15) was configured to determine *f*_B_, which was fed to a second Morlet wavelet of high time resolution (*N*=3) and central frequency *f*_B_ for convolution with the measured signal in order to trace the time evolution precisely at *f*_B_,, thereby providing an accurate extraction of *τ*_0_.


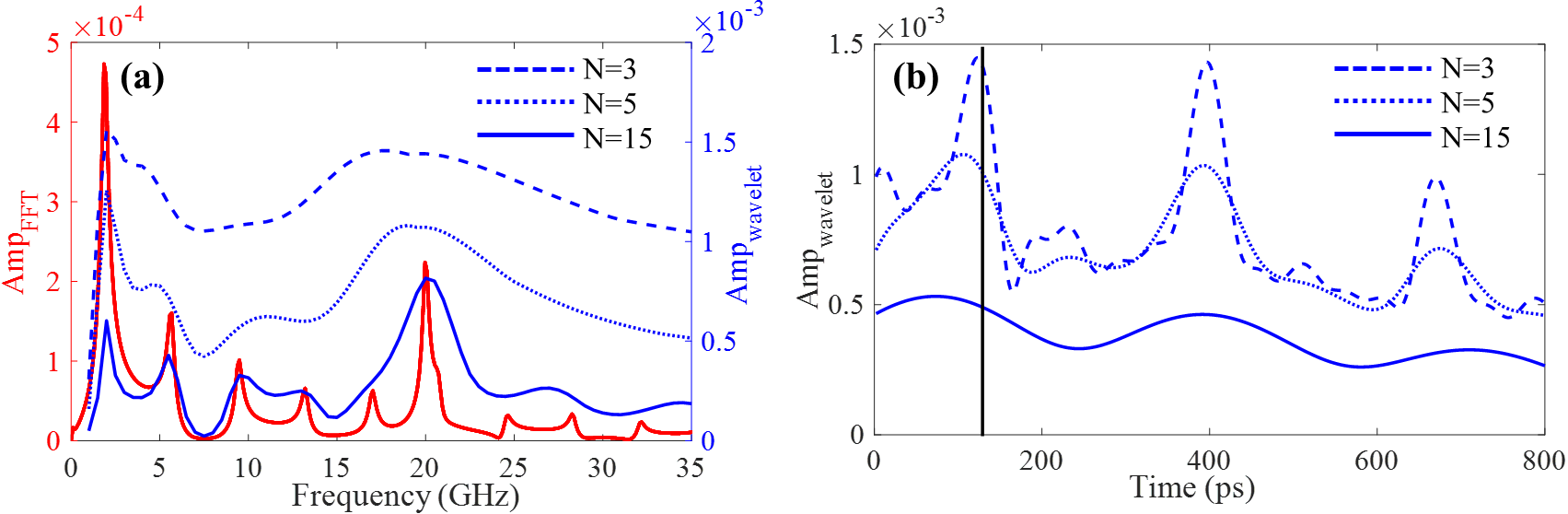


***Figure S2*** *Trade-off between the temporal and frequency precisions. (a) The frequency spectrum obtained by the Morlet transform at different N values, i.e. vertical lines in Fig. S1, are presented in the right axis and compared with the FFT spectrum of the simulated signal, in red, shown in the left axis. When N=15 is configured, the frequency components of interest can be identified correctly, complying with peaks in FFT spectrum (left axis).* *(b) The convolutions of the simulated reflectivity change with a wavelet of central frequency f_B_ are plotted for different N values. When N=3 is configured, the time location of τ_0_ can be determined precisely, with ~3 ps offset compared to the expected value, marked by the vertical line in (b).*

**Experimental set-up**

In this work, the all-optical tracking of CAPs was carried out using the setup shown in Fig. 2. A compact dual-oscillator (t-Pulse Duo, Amplitudes Systemes®), containing two passively mode-locked Yb:KYW laser cavities, emits two trains of laser pulses (400 fs) with one serving as pump (1030 nm) and the other as probe (515 nm). The two laser pulses are operated at approximately *f*_0_ = 48 MHz, with a slight repetition frequency shift, Δ*f*, to perform asynchronous optical sampling (ASOPS).^3^ In this work, *Δf* is generated by a frequency synthesizer and is stabilized at 2 kHz using an active feedback loop. In such a configuration, a time delay of ~20 ns, reciprocal of *f*_0_, is scanned within 500 μs, reciprocal of Δ*f*, with time resolution of 870 fs, without involving any mechanical motion. Hence it avoids several intrinsic drawbacks encountered in the conventional mechanically-controlled delay scanning, for instance the fluctuation of the beam pointing, modification of the size of the focal spot on the sample, and long acquisition time. The two beams are coaxially aligned and focused by an objective lens, x50 (beam dia.=1.0 µm), through a sample (cell) onto the Ti plate surface, which is situated on a translation stage (PI5633D, Physik Instrumente®), thus allowing the 1D and/or 2D scanning of the sample. At the sample/Ti interface, each pump pulse launches a longitudinal strain pulse, which alters the intensity of the reflected probe light during its propagation, as described in Section II. The resulting intensity variation of the probe light is monitored by a photo-detector (PDB440A, Thorlabs®) in connection with a 14-bit digitizer (PXI-5122, National Instruments®). Data readout and averaging is done with homemade Labview (National Instruments®) programs installed on a computer which communicates with the digitizer via a GPIB interface. While measurements were being taken, low pump and probe power, 3 mW and 1 mW, respectively, were used to avoid any possible photo-damage to the cells. The power was measured with a power meter (1917-R, Newport®) placed just beneath the objective lens. Thanks to the high acquisition speed of the ASOPS configuration, this setup is capable of determining in a single measurement optical reflectivity changes as small as 10^-5^ in approximately 5 seconds. This yields 12 pixels per minute in a raster scan fashion, typically 4 hours for mapping 50×50 µm^2^ with 1 µm lateral resolution. It should be noted that such mapping produces straightaway a movie of interest of 2 ns duration, (maximum 20 ns determined by the repetition rate of the laser, however the signal of interest is generally around a few nanoseconds or less) with 870 fs per frame, *i.e.*,~2000 frames in total. The acquisition time for a single frame is therefore around 7 seconds, comparable to that for state-of-art high resolution microscopy techniques, including confocal microscopy, stochastic optical reconstruction microscopy, and photoactivated localization microscopy. Another notable feature of this setup is its high compatibility with other commonly used microscopic methods. In this setup, the main apparatus involved in the PU detection is accommodated in a commercial reflected light microscope (BXFM-F, Olympus®), as shown in the shaded area in Fig. 2. By translating the switchable mirrors allocated between the objective nosepiece and the filter cube, it is convenient to switch the function of the setup between PU study, using the 100% reflection mirror, and reflected field microscopy, using the 50% or the 100% transmission mirror. This allows a precise alignment of the laser beams onto a target sample (cell), but also gives convenient access to other microscopic knowledge of the sample (cell) under inspection. Furthermore, in this setup PU detection can also cooperate with reflected fluorescence microscopy by using the installed filter cubes, and this is of particular benefit for performing a joint study over regions of interest (ROI) marked with fluorescent labels.

**Spin coating**

A mixture of PMMA and chlorobenzene was first prepared by dissolving 150 mg of PMMA granules (Mw~50000, Sigma Aldrich) in 1 ml of chlorobenzene at room temperature. The mixture was then stirred for approximately 3 hours at 50° Celsius until a clear homogeneous and transparent matrix formed, which served as the coating solution in this work. The spin coating fabrication of thin PMMA films was performed on commercially available Ti substrates by using a spinner (RC8, Suss Microtec). Each Ti plate was cleaned thoroughly before use with acetone and methanol, followed by a deionized water rinse and N_2_ blow-dry in order to remove small particles and organic contaminations on the surface. By running the spinner at 500 rpm and 3000 rpm for 20 seconds, thin films of thickness 1800 nm and 150 nm were deposited on the Ti substrates respectively, measured using a stylus profilometer (Alpha-Step IQ, Kla-Tencor®). Before the thickness measurement, the substrates were kept at 80° Celsius overnight to evaporate the solvent in the film.

**Cell culture and fixation**

- **Osteosarcoma cells**

HTB96 U2OS cells (ATCC) were cultured in McCoy’s 5A medium (Dutscher) supplemented with 10% (v/v) Fetal Bovine Serum (FBS, Dutscher) and 100 µg/ml streptomycin/penicillin (Pen-Strep 15140, GIBCO). HTB96 U2OS cells were seeded on the surface of EtOH-sterilised Ti plates for incubation overnight and fixed with paraformaldehyde 4% (w/v) in phosphate-buffered saline medium (PBS, pH 7.4, without Ca^2+^ and Mg^2+^) for 15 min at room temperature and rinsed with PBS. Samples were maintained in PBS at +4°C.

- **Macrophages and Monocytes**

Human monocytic THP-1 cells were maintained in culture in Roswell Park Memorial Institute medium (RPMI 1640, Sigma) containing 10% of heat-inactivated fetal bovine serum (Invitrogen). THP-1 monocytes are differentiated into macrophages by 24h incubation with RPMI 1640 medium containing 150 nM phorbol 12-myristate 13-acetate (PMA, Sigma, P8139) followed by 24h incubation in RPMI medium. Monocytes and macrophages were seeded on the surface of EtOH-sterilised Ti plates for incubation overnight. Cells were then washed with PBS and fixed by PFA (4%) at 4^o^C for 15 minutes. Two samples were rinsed with water to prevent formation of salt deposits and left to dry in air.

**AFM mapping**

The morphology of the osteosarcoma cell was also studied using a commercial AFM instrument (Dimension 3100, Bruker) in tapping mode at room temperature. TESP/TESPA (Bruker) cantilevers with a nominal spring constant of 42 N m^-1^ and a resonance frequency of 320 kHz were used in AFM mapping. The cantilevers are 125 nm in length and 40 nm in width, and have a silicon pyramidal tip with a radius of curvature of 8 nm.

**PU and AFM mapping of an osteosarcoma cell**

Two routes were combined for thickness measurement in order to reconstruct the whole 3D morphology of the cell from the recorded movie (Online movie 1). The cell nucleus is indeed generally thick, a favourable situation for Brillouin interaction detection, while the cytoplasm can be very thin, favourable for acoustic resonance detection. Fig. S3 shows two representative waveforms measured in the cell nucleus (a) and in cytoplasm, respectively (b). They show a high signal to noise ratio (SNR) allowing accurate determination of *f_B_*, *τ*_0_ , Fig. S3(a), and *f_R_*., Fig. S3(b).


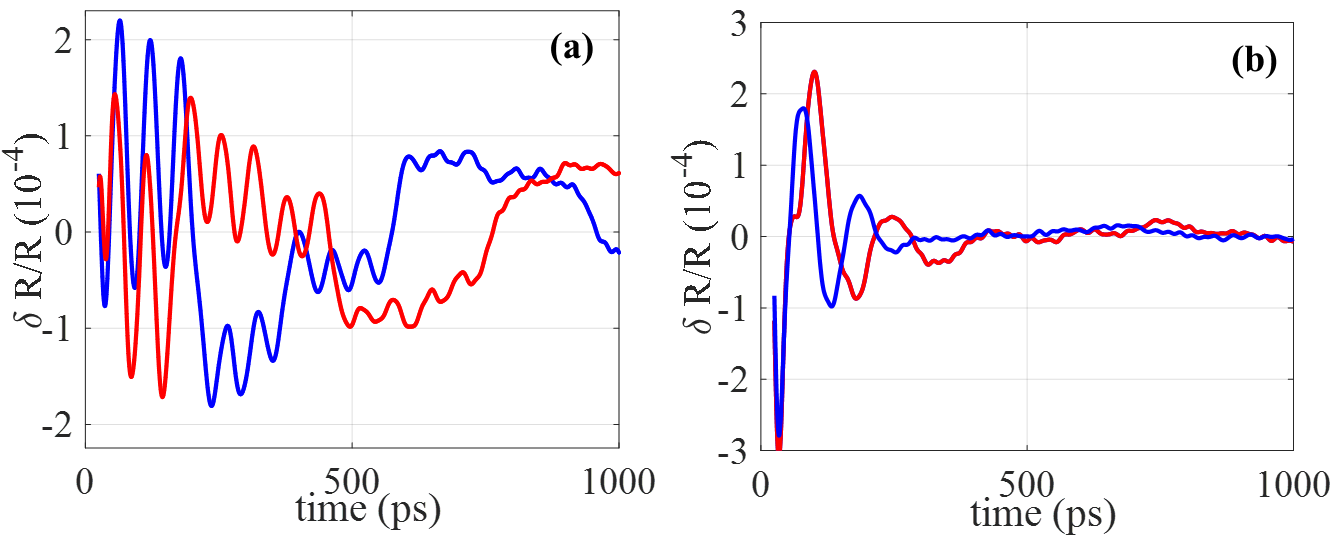


***Figure S3*** *Typical waveforms recorded for the osteosarcoma cell. Two representative waveforms measured in nucleus (a) and cytoplasm (b). The signals show high SNR, allowing accurate measurements of f_B_, τ_0_ or f_R_.*

The stages for thickness mapping of the whole cell are illustrated in Fig. S4. For each pixel in the thick nucleus, we measured both Brillouin frequency (a) and *τ*_0_ (b) by using the aforementioned time-frequency analysis. From *f*_B_ one can calculate the sound velocity (c) with $v=\lambda f_{B}/2n$ assuming constant value for optical refractive index, 1.35 in this work. The thickness can thereby be obtained simply by $L=vt_{0}$ for each pixel (d). In the cytoplasm, we measured the acoustic resonance frequency $f_{R}$ (e) for each pixel, and calculated the thickness (f) with $L=v/4f_{R}$. We used *v* = 3.5 nm/ps as the mean value of the sound velocities measured in the thick part of the cell, shown in Fig. S3 (c).


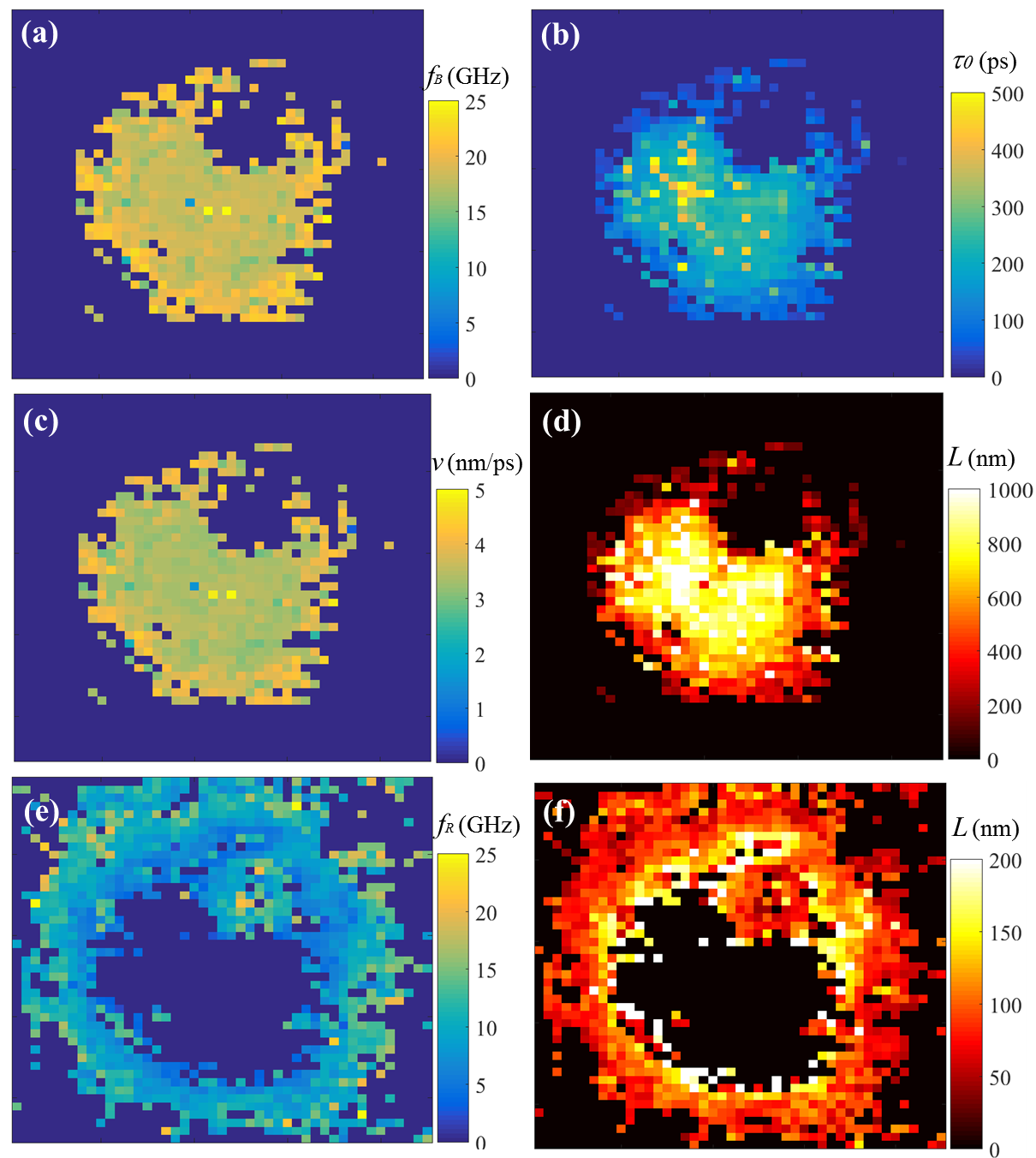


***Figure S4*** *Complete data processing for thickness mapping of the entire cell. A combined application of the two routes for thickness measurement was used to reconstruct the whole thickness map. In the nucleus f_B_ (a) and τ_0_ (b) were measured at each pixel. The sound velocity v (c) was calculated from f_B_ with a fixed value of the optical refractive index (1.35). The thickness map in the nucleus (d) can then be obtained with $L=vt_{0}$. In the cytoplasm the acoustic resonance (e) was used to map the thickness (f) with L=v/(4f_R_), considering the mean velocity value in (c), v= 3.5 nm/ps.*

Thickness mapping of the same osteosarcoma cell was performed with an AFM for the purpose of comparison. The data measured in a square of 60×60 µm^2^ (512 × 512 pixels) are shown in Fig. S5 (a). The raw data for height given by the AFM were then converted into a thickness map, as shown in Fig. S5 (b). For this, the mean height value measured in the bare titanium, -146 nm, was subtracted. Next, a rotation matrix, 45 degrees counter-clockwise, was applied for a straightforward comparison with the AFM image, Fig. S5 (c), with the cell 3D morphology measured remotely using the PU technique. It can be seen that the two techniques provide the same cell morphology. A quantitative analysis of both images is given in the bulk of the manuscript.


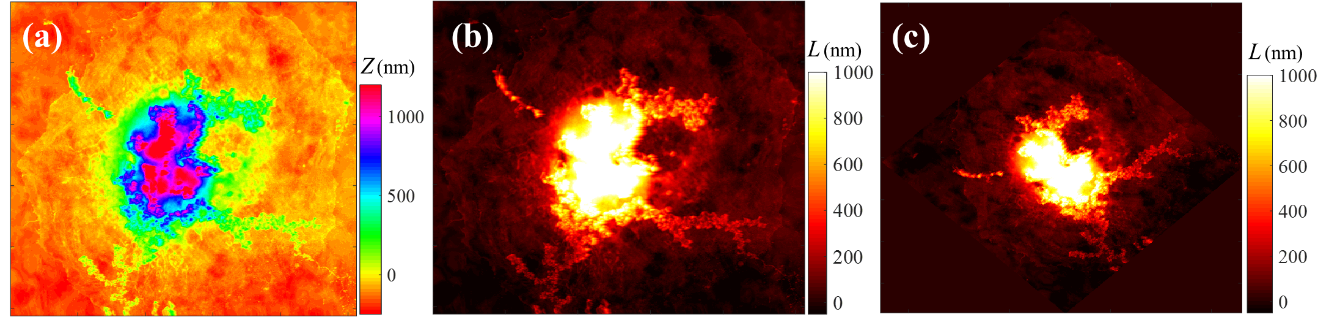


***Figure S5*** *Processing of the AFM data. The raw data are the height (a) of the osteosarcoma cell in a square of 60×60  µm2. By subtracting the mean height value at the bare Ti part, -146 nm, one can obtain the thickness map (b). A further rotation matrix, 45 degrees counter-clockwise, was applied in order to have the same cell orientation (c) as in the PU measurement for the sake of convenient comparison.*

For further illustration the thickness maps measured by the two techniques are shown in 3D plots given in Fig. S6. They confirm that the two techniques give similar cell shape and cell height mapping. This comparison shows that although the in-plane resolution is less, the remote PU technique is convenient for revealing the roughness of the osteosarcoma cell.


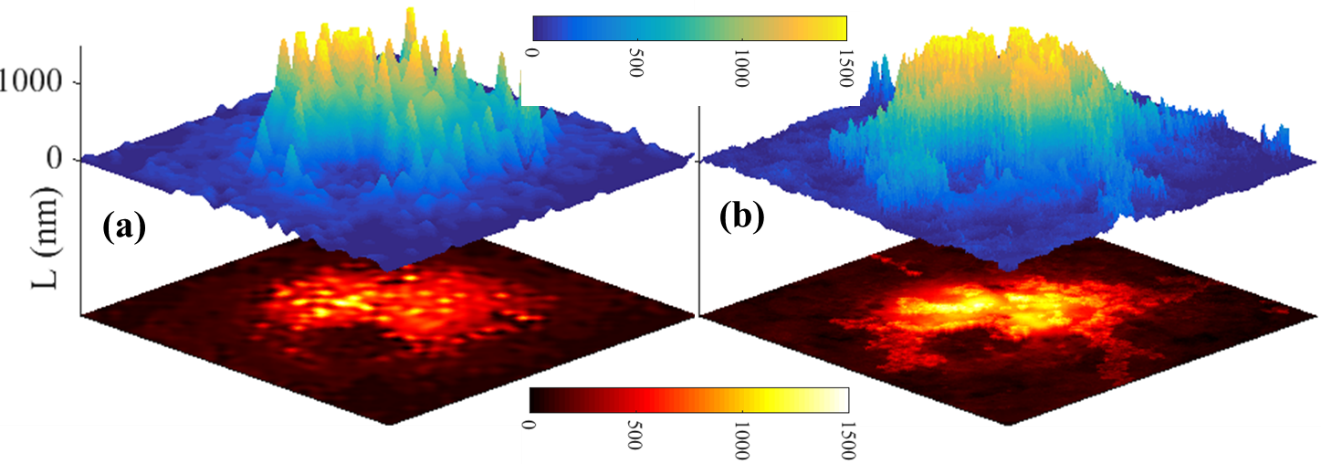


***Figure S6*** *3D representation of the measured cell morphology. A comparison of the 3D cell morphology measured by means of the PU (a) and AFM technique (b). A 2D spine interpolation was applied to the PU map for better illustration.*

**Effect of inhomogeneity of cell physical properties on thickness imaging with PU**

In this section we analyse the sensitivity of the thickness measurement with PU to cell inhomogeneity. We discuss the effect of inhomogeneity, either along the in-depth direction *z* or along the scanned (*x*,*y*) plane, of the cell mechanic properties, sound velocity or mass density, and of the optical index.

In the thick part of the cell, we measure the sound velocity in cell from the recorded frequency of the time resolved Brillouin interaction $f_{B}=2nv/\lambda$. The time resolved oscillations result of the scattering of the probe light by the acoustic wavefront as long as the latter propagates through the cell. This frequency does not change significantly with time in the recorded time traces, ie with the propagation of the acoustic wavefront along depth. This means that the sound velocity is constant with depth at the scale of the phonon wavelength, ie $\lambda_{B}\approx200\text{ nm}$. This velocity is that in a material with mechanic (stiffness, mass density) and optical (refractive index) properties averaged along one phonon wavelength. The frequency measurement is insensitive to inhomogeneities at smaller scales, although sensitive to the nucleus homogeneous nano-structuration at that scale[2]. The thickness measurement with PU in the thick part of the cell is thus insensitive to inhomogeneity along *z* of size $<<\lambda_{B}$. In the (*x*,*y*) plane, since sound velocity is measured at each pixel, the thickness measurements account for possible sound velocity inhomogeneity in the (*x*,*y*) plane. Such inhomogeneity does not bias our measurement. However, the cell refractive index, which we did not measure, had to be assumed constant from one pixel to another. The refractive index changes inside a single cell are small, by 4% only.[4] Possible effect on our measurements is discussed in the article with the analysis of the experimental results shown in Figs 3, 4 and 6.

In the thin part of the cell, the thickness is calculated with the measured resonance frequency assuming sound velocity. Again the material is considered homogeneous with averaged (homogenised) properties over thickness. Since the film behaves as an acoustic quarter wave plate the wavelength at the resonance frequency we are considering is by four fold larger than the thickness. The actual wave velocity, thus the resonance frequency is insensitive to inhomogeneities smaller than this wavelength. The measured cell thickness is thus insensitive to small mechanic or optical inhomogeneities. In this thin part the actual averaged velocity cannot be measured. We thus had to consider the mean value of the sound velocities we measured with Brillouin at several pixels in the thick part of the cell. This may introduce a bias in the thickness measurement of the thin parts. To give an idea of this possible bias amplitude, dispersion of the sound velocities we measured in the thick part of the osteosarcoma cell is of 10 %. If the dispersion of sound velocity in the thin parts of the cell, the lamelipodium, is of same order then assuming a constant value can induce an error by 10 % of the thickness measurement. This can explain the small differences between AFM and PU data in the thin part of the cell profile we imaged in Fig. 6(c).

1. M. Ducousso, O. E.-F. Zouani, C. Chanseau, C. Chollet, C. Rossignol, B. Audoin, and M.-C. Durrieu, Eur. Phys. J. Appl. Phys. **61**, 11201 (2013).
2. O. F. Zouani, T. Dehoux, M.-C. Durrieu, and B. Audoin, Soft Matter **10**, 8737 (2014).
3. A. Abbas, Y. Guillet, J.-M. Rampnoux, P. Rigail, E. Mottay, B. Audoin, and S. Dilhaire, Opt. Express **22**, 7831 (2014).

4 K. G. Phillips, S. L. Jacques, and O. J. T. McCarty, Phys. Rev. Lett. 109, 118105 (2012)
